# Commentary: Predictors of Colorectal Cancer Screening in Two Underserved U.S. Populations: A Parallel Analysis

**DOI:** 10.3389/fonc.2020.00240

**Published:** 2020-03-05

**Authors:** Roman Johnson, Jennifer R. Bail, Michael Behring, Rachael Orlandella, Victoria Williams, Karina I. Halilova, Teri W. Hoenemeyer

**Affiliations:** ^1^Department of Sociology, University of Alabama at Birmingham, Birmingham, AL, United States; ^2^Department of Nutrition Sciences, University of Alabama at Birmingham, Birmingham, AL, United States; ^3^Department of Pathology, University of Alabama at Birmingham, Birmingham, AL, United States; ^4^Graduate Biomedical Sciences, University of Alabama at Birmingham, Birmingham, AL, United States; ^5^Department of Health Behavior, University of Alabama at Birmingham, Birmingham, AL, United States; ^6^Division of Preventive Medicine, Department of Medicine, University of Alabama at Birmingham, Birmingham, AL, United States

**Keywords:** colon cancer incidence, colon incidence hotspots, spatial cancer, interdiscipinarity, gut micobiome

## Introduction

The authors contribute to the literature on geographic colorectal cancer disparities by examining two different populations located in the major colorectal cancer incidence (CRC) hotspots of the Lower Mississippi Delta, West Central Appalachia, and Eastern Virginia/North Carolina regions (1). However, this study could be improved by incorporating more measures reflective of the neighborhood deprivation literature, such as fruit and vegetable consumption (which may decrease colon cancer risk) and extensively underscore the role of the gut microbiome in colon cancer risk (2–5). For example, fruit and vegetable consumption has been shown to reduce the risk of colon cancer (6, 7). In one study, fruit consumption and only one vegetable category, legumes, was inversely associated with colon cancer risk (6). Another study found that consumption of green and white vegetables and fruits was inversely associated with colon cancer risk in men. Simultaneously, consumption of green, red/purple, and white vegetables and fruits was inversely associated with colon cancer risk in women (7). However, in the same study, consumption of orange/yellow vegetables and fruits in men was associated with increased colon cancer risk (e.g., citrus fruits and ginger) (7). Furthermore, diversifying the gut microbiome can aid in preventing colon cancer (8). For instance, eating red meat—a known carcinogen and an inflammatory agent in the bowel—is a common activity in the United States, and largely so in the Midwest and Deep South (8, 9).

## Geographical Differences of Colorectal Cancer Hotspots

While there was elaboration on the characteristics of CRC hotspots, the characteristics of CRC non-hotspot areas were not as detailed. For example, the study may have benefited from communicating how cancer incidence and mortality varies within the CRC non-hotspot and hotspot areas (10). Secondly, the fact that some men may hold machismo health beliefs (such as the belief that they can delay healthcare because of their capacity for pain) is an important health belief consideration for explaining the observed differences between men and women in CRC (11). For men, hyper-masculinity ideals may delay or deter them from seeking preventive healthcare services, and, in particular, colonoscopy (11).

In a hotspot-originating analysis, mortality rates for African-American (AA) males in the Lower Mississippi Delta Region (comprising 94 counties across Arkansas, Illinois, Kentucky, Louisiana, Mississippi, Missouri, and Tennessee) have remained unchanged, while all other race/gender groups have declined in the last 25 years (12). In this study, traditional risk factors such as AA race and unemployment were linked to higher adjusted relative odds of being *within* screening guidelines. Interestingly, this gender disparity (38% males within guidelines), is seen in screening in the Southern Community Cohort (SCCS) study, which comprised the subject of this commentary (1, 13).

## Opportunities for Interventions

While the authors highlighted the importance of future interventions in underserved areas to increase CRC screenings, specific recommendations regarding how the study's findings translate into specific health behavior change interventions were not provided. Lack of health insurance, low income, and smoking are well-documented colon cancer risk factors screening as well as viable opportunities for preventative intervention (14–16). Furthermore, the Community Guide recommends multicomponent interventions to increase screening for colorectal cancer, and the National Cancer Institute (NCI) provides a list of research-tested intervention programs (RTIPs) specifically aimed at increasing colorectal cancer screening (17, 18). For example, Targeting Cancer in Blacks (TCiB) is a multicomponent, community-based intervention focused on awareness building and behavior modification in unscreened and under-screened AA adults (19). This 18-month intervention, which was disseminated through historically black colleges and universities (HBCUs), resulted in increased cancer screening among AAs in the Southeast United States. An intervention in a rural Washington state community of Hispanic individuals found that use of a *promotora* cultural worker increased colon cancer screening awareness, knowledge, and screening use in that population (20).

The finding that two out of the three CRC hotspot regions exhibited increased odds of CRC screening is intriguing. However, it is also possible that biological differences in CRC severity in hotspot regions exacerbated by race/ethnicity, lower educational attainment, higher obesity prevalence, and unhealthy lifestyles may lead to increased colorectal cancer mortality (21–25). Therefore, future studies should examine potential biological differences in CRC patients who reside in hotspot and non-hotspot areas to improve patient care. Specifically, the potential differences in the gut microbiome matter for individuals at greater risk of colon cancer, especially for those who occupy multiple disadvantaged social profiles and those whose typical diet is more fatty in composition (e.g., black individuals who live in urban environments concentrated with fast food restaurants and low quality produce in grocery stores) as obesity increases colon cancer risk.

Health professional recommendations have a positive influence on CRC screening (26). A pivotal role in patient education and motivating screening adherence is played by nurses, given the extended time they spend with patients. Arnold et al. (27) conducted a nurse-led intervention aimed at increasing knowledge and self-efficacy for CRC screening with Fecal Occult Blood Test (FOBT). Nurses used motivational interviewing techniques to identify, solve barriers, and motivate patients to complete FOBTs. Among those receiving nurse support, self-efficacy increased significantly, with patients indicating they could obtain an FOBT, complete it and mail in results. Thus, future interventions aimed at improving CRC screening adherence among those residing in hotspot regions should consider a nurse-led approach.

In conclusion, we are heartened by the contribution of Bernardo et al. as it further elucidates our understanding of screening differences by geographical regions. By building further on this research, with an understanding of how to link efficacious interventions, multidisciplinary efforts can improve the health outcomes of the regions' underserved populations. Additionally, research that focuses on geographical screening differences should be translatable into an interdisciplinary, behavioral-based intervention improving health outcomes.

## Summary of Risk Factors for Colon Cancer

- Lifestyle and Diet (28, 29)
- Personal History and Genetics (21, 30)
- Racial/Ethnic Background (31, 32)
- Geographical Environment (33, 34).

## Author Contributions

Each member of the study team contributed portions of the earlier draft manuscripts. The corresponding author prepared the final draft of the manuscript and edited earlier versions of this manuscript.

### Conflict of Interest

The authors declare that the research was conducted in the absence of any commercial or financial relationships that could be construed as a potential conflict of interest.
